# The Imperfect World of Global Health Estimates

**DOI:** 10.1371/journal.pmed.1001006

**Published:** 2010-11-30

**Authors:** Peter Byass

**Affiliations:** 1 Umeå Centre for Global Health Research, Department of Public Health and Clinical Medicine, Umeå University, Umeå, Sweden; 2 Immpact, University of Aberdeen, Aberdeen, United Kingdom

## Abstract

Peter Byass provides an introduction to a PLoS Medicine cluster of articles on global health estimates, and argues why the "estimates debate" is so important.

**Summary Points**

- Global estimates of population health are currently needed because of the shortage of adequate quality population-based data. These estimates are complex, because they need to combine relatively complete data from industrialised countries with sometimes very scanty data from developing countries.
- Two major sources of such estimates are agencies within the United Nations system and (mostly northern) academic institutions, which differ in their approaches.
- Appropriate strategies for ensuring the robustness and transparency of estimates are very important. Long-term strategies must be geared towards improving the quantity and quality of bottom-up data, rather than developing ever more complex estimation methods.
- Ultimately, the world must be able to measure population health from reliable individual data rather than relying on estimates.

*This article is part of a cluster of five articles on global health estimates.*

## How Did We Get Here?

Global measurement has long been contentious. Three hundred years ago, the exact size and shape of the world were a matter of scientific controversy and estimation. New ways of measurement were developed, and by the early 18^th^ century that uncertainty ceased [1]. One hundred years ago, Sweden started to account for all deaths in its population, by cause, age, and sex [2], and annual summary tables based on individual death registration on a national basis have been published ever since. Today, despite increasing globalisation, there is still no similar universal individual registration of vital events in many countries, and consequently we find ourselves in an era of global estimates of population health. These global estimates are complex amalgams of detailed national measures from countries with universal registration and the best available data—which are often scanty—from other settings. Hopefully the long-term aim of the global health community is to move beyond this era of estimates, towards the relative certainty of accounting for individual health globally. Meanwhile, the purpose of this article is to explore issues and tensions around these currently necessary global estimates.

As discussed previously [3], fundamental links between poverty and data availability mean that global estimates are not trivial to construct. Unlike global estimates of geophysical parameters, which have been revolutionised by remote sensing and satellite surveillance [4], population health estimates must continue to rely in some way on grass-roots data about individual people. But lack of standards and different approaches are causing contention and confusion.

## Why Is There Debate about Current Global Estimates?

**Figure 1:**
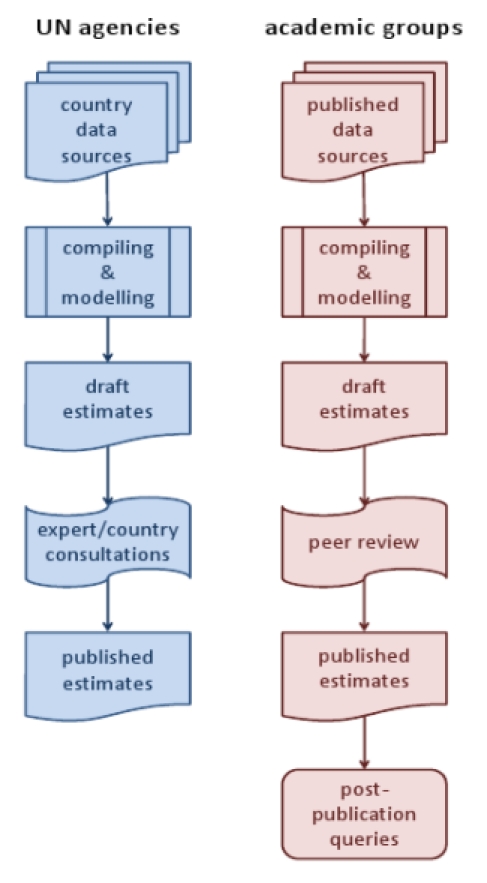
Approaches to global estimates by UN agencies and academic groups.

Current global estimates mainly come from one of two sources: (1) the United Nations (UN) and its specialised agencies (such as the World Health Organization and the United Nations Children's Fund [UNICEF]) or (2) northern academic institutions. There are important underlying differences between the estimates from these sources, as shown in Figure 1. Why southern academic institutions are not more engaged in the process of developing global estimates, given that the major uncertainties within most estimates centre on southern data, is a further question of interest.

### The UN Estimates

To contextualise estimates originating from the UN, it is necessary to understand the nature of the UN system. The UN is fundamentally constructed as a member organisation for most of the world's nation states, and member countries are therefore its constituency. This structure has significant practical implications for the construction of global estimates. The UN system has a direct entrée to member countries at the governmental level and, although unable to exercise compulsion, may be able to access otherwise unpublished national data. After UN experts have processed and modelled available data, member countries are commonly consulted on estimates before publication. UN estimates can then be published under the UN imprimatur [5], although often staff and external advisors also prepare articles for publication in peer-reviewed journals [6]. This approach has been criticised [7], perhaps unfairly.

### Estimates from Academic Institutions

Academic institutions, by contrast, have to rely largely on public-domain data and previously published studies, often leading to the use of mixed methods involving direct and meta-analyses [8]–[10]. As independent institutions, they have no obligation to consult externally, and countries' first sight of such estimates may well be on publication. While this may lead to interesting post-publication debate and controversy, there can be a problem in that a published paper is still likely to be taken as the definitive version, disregarding any implications of subsequent interactions. Such estimates are, however, normally disseminated via peer-reviewed journals, which should be assumed to assure a paper's quality, as with any other scientific output. However, questions have been raised as to how to effectively peer-review papers describing complex estimates [11]. An interesting development might be the inclusion of a country consultation phase before publication of estimates from academic institutions, making public the issues that thereby arise.

## Two Tales of Maternal Mortality

Some of the recent debate and contention around the source of global estimates emerged during 2010 when two separate estimates of global maternal mortality were published. One set of estimates originated from the Institute for Health Metrics and Evaluation (IHME) [12], and the other was the latest update of the UN inter-agency estimates of maternal mortality [13]. It is impossible to conclude which is the more “correct” set of estimates, because if that were measurable as a matter of fact, the estimates would be redundant anyway. However, it is interesting to compare some key issues in the approaches and conclusions of these different estimates. Headline figures were very similar and well within each other's uncertainty intervals (342,900 and 358,000 maternal deaths worldwide and maternal mortality ratios of 251 and 260 per 100,000, respectively, for 2008). However, there may be important differences at the country level or for specific causes, depending on the data and methods used.

On the critical issue of estimating how many deaths among pregnant women might be associated with HIV/AIDS, very different approaches were used, with widely different conclusions (61,400 and 42,000 deaths, respectively, for 2008). Bottom-up data on deaths associated with both pregnancy and HIV/AIDS—particularly from Africa—are very scanty because, even where verbal autopsies are performed to ascertain cause of death, very often only a single cause is recorded [14]. It is not surprising that where data are critically lacking, different approaches to estimation yield different results. The overlap between pregnancy and HIV/AIDS is an area in which urgent reforms in methods and procedures are needed in order to provide appropriate multiple-cause data, rather than developing more complex estimation methods.

## How Can the Robustness and Transparency of Global Estimates Be Ensured?

Because estimates are estimates, and not measurements, it is relatively easy for the proponents of particular estimates to claim high quality and reliability, and for the detractors to question the same, with little scope for objective adjudication. The key factor for robustness is the extent of available data, linked, of course, to sound methods. Transparency involves using all available data of quality and relevance, while usually imposing some explicit framework of rules as to what constitutes usable data. Methodological strategies then also need to be set out in a fully transparent manner. Any epidemiological interpretation depends on an understanding of the provenance and sampling basis of the underlying data, which leads to estimates of uncertainty. However, some of the data used in global estimates are so many stages removed from their origin that associated estimates of uncertainty themselves become very complex and hard to understand. Unfortunately, the gaps and uncertainties around data in many instances drive researchers to ever-increasing levels of methodological complexity in attempts to compensate, and transparency may be obscured by these complexities. This can rapidly lead to an “Emperor's New Clothes” syndrome in which only the cognoscenti truly understand the underlying basis of complex estimates, while the vast majority may be reluctant to admit that the detail is beyond their comprehension.

## Where Do We Need to Go from Here?

The undeniable long-term aim must be to foster more and more production of high-quality and complete population data from locations that are as yet devoid of usable material. This implies a bottom-up philosophy emphasising the need to connect with population data at source [15]. If a gradual process of filling in such gaps in global data is realistic, then one would also hope that in parallel with increasing completeness of data there will be reductions in the complexity of appropriate estimation methods. This would lead towards the ideal situation, in which global estimates would become a thing of the past as the world's population actually became measurable.

One potential obstacle to this process is that the world may become so used to the concept of global estimates that insufficient effort will be invested in improving bottom-up data. Even now, there are signs that journal editors can find complex global estimates more enticing to publish than relatively detailed epidemiological descriptions of within-country data. For example, four recent sets of global estimates published in *The Lancet* have been “fast-tracked” for reasons that are not entirely clear [10],[12],[16],[17]; since they actually present long-term estimates that have no urgent health care implications, perhaps this is symptomatic of the self-importance that such estimates seem to be acquiring. It is very important that the concept of global estimates does not acquire an undeserved supremacy over conventional analyses of detailed data, if we want to nurture a culture of encouraging and promoting good-quality data at source, with local analyses and interpretation [18]. Strategies to bring all countries to a common standard of high-quality and sustainable health information systems need to be prioritised [19].

For the foreseeable future, the global health community is likely to be locked in a love–hate relationship with global estimates of population health. We wish we did not need these estimates and could instead rely on objective assessments based on quality data. But we know we do not yet have enough of those data, and so for now we do need estimates as a resource on which to base health policy and planning decisions. However, we should not expect to wait three hundred years, nor even one hundred years, before superseding global estimates with global measurements.

**Global Health Estimates: Where Do We Go from Here?**

- Work towards better availability and quality of data
- Enhance country capacity to develop, analyse, and interpret local data
- Develop common data standards
- Focus on better data rather than more complex estimation methods
- Improve robustness of data and methodologies used in estimates
- Manage a gradual transition from estimates to measurements
